# Endovascular Repair in Blunt Thoracic Aortic Injury: A 10-Year Single Center Experience

**DOI:** 10.7759/cureus.55327

**Published:** 2024-03-01

**Authors:** Hanan Edah AlQurashi, Hassan Ahmed Alzahrani, Mohammad Omar Bafaraj, Mohammed Bosaeed, Mohammed Almasabi, Abdulrahman Banhidarah

**Affiliations:** 1 Vascular and Endovascular Surgery, Alnoor Specialist Hospital, Makkah, SAU; 2 General Surgery, King Abdulaziz Hospital, Makkah, SAU

**Keywords:** thoracic, repair, injury, endovascular, blunt, aortic

## Abstract

Background: Life-threatening blunt thoracic aortic injury (BTAI) typically occurs alongside multiple other traumatic injuries. Symptoms of BTAI can range from being asymptomatic in the case of intimal tears to becoming catastrophic in the case of uncontained aortic ruptures. The aim of this research was to examine the clinical outcomes for those who underwent thoracic endovascular aortic repair (TEVAR) in hospital settings.

Methods: A cross-sectional retrospective study was conducted using patient data that were extracted from Al-Noor Specialist Hospital, Makkah, Saudi Arabia, for the duration between January 2011 and December 2021. This study included data from all patients aged 18 and up who had been diagnosed with BTAI and had undergone TEVAR. The BTAI diagnoses were confirmed using CT scans. Logistic regression was utilized to identify predictors of patients’ health status improvement and length of stay.

Results: A total of 80 patients were involved. Around 50.0% (n=40) of the patients had grade 3 thoracic aortic injuries. The median duration of stay was 14.00 days (Interquartile range 21.00). Only one patient developed post-procedure complications (1.3%). Almost one-third (31.3%; n=25) of the patients required subclavian coverage. One patient developed intraoperative endoleak (1.3%). One patient developed an access site complication (1.3%). The mortality rate within 30 days of the operation was 1.3%. The vast majority of the patients (92.5%; n=74) showed improvement upon discharge from the hospital. The baseline patient characteristics and length of hospitalization had no effect on the improvement of patient status upon discharge or their length of stay (p>0.05).

Conclusion: Patients with BTAI have shown an excellent success rate with TEVAR and a low complication rate. Predictors of procedure success and length of stay need to be identified; however, this can't be done without larger-scale investigations. This can aid in the development of preventative measures that improve clinical outcomes for the patients.

## Introduction

Blunt thoracic aortic injury (BTAI) is a life-threatening situation frequently accompanied by several traumatic injuries [1]. The presentation of BTAI varies significantly from asymptomatic intimal tears to devastating uncontained aortic ruptures [2]. BTAI is linked to a high mortality rate [1]; the second most prevalent reason for blunt trauma mortality, after head injuries, is BTAI [3,4].

The most typical situation for BTAI is a sudden deceleration, as in a crash injury, a fall injury, a motorcycle crash, or a car crash [3]. About 80% of BTAI patients pass away before being admitted to the hospital, with in-hospital mortality reaching 46% for the remaining survivors [5-7]. Each year, more than 1,100 hospital admissions are reported in the United States of America (USA) due to BTAI [8].

The traditional treatment for BTAI has been open surgical repair, even though this procedure is linked to a paraplegia rate of 16% and a mortality rate of 28% in the trauma setting [9]. However, over the past two decades, there has been a remarkable transformation in the management of surviving hospital admission patients as a result of technological advancements in diagnosis, including the availability of CT, early control of blood pressure [10], and the use of thoracic endovascular aortic repair (TEVAR) [11].

In 1997, the first description of TEVAR's use for BTAI treatment by custom devices emerged [12]. Subsequently, these devices received FDA approval in 2005 and are nowadays available in various sizes. As a result, the dominant method for repairing injured aortas has become TEVAR [13-15].

According to the clinical practice guidelines of the Society for Vascular Surgery (SVS) recommendations, TEVAR exhibits a reduced incidence of paraplegia compared to surgical graft replacement of the aorta [3,4,13,16,17]. Additionally, it is linked to higher survival rates compared to non-operative or open repair management [3,4,13,16,17]. Thus, several studies in Western countries have linked the overutilization of TEVAR over open repair and the improvement in patients’ outcomes [15,18,19]. However, several issues remain unanswered, and there are limited studies on the clinical outcomes of patients who underwent TEVAR within hospital settings.

The aim of this study was to explore the clinical outcomes of patients who underwent TEVAR in hospital settings. Specifically, we aimed to explore the technical success rate, post-procedural complications, and patients’ mortality within 30 days.

The research questions consisted of the following. What were the clinical outcomes of patients who underwent TEVAR in hospital settings? What was the technical success rate of patients who underwent TEVAR? What were the post-procedural complications of patients who underwent TEVAR? What was the mortality rate within 30 days for patients who underwent TEVAR?

## Materials and methods

Study design

A cross-sectional retrospective study was conducted using patient data that were extracted from Al-Noor Specialist Hospital, Makkah, Saudi Arabia, for the duration between January 2011 and December 2021. 

Study population

Inclusion and Exclusion Criteria

This study included data from all patients aged 18 and up who have been diagnosed with BTAI and have undergone TEVAR. BTAI diagnoses were confirmed using CT scans. All patients who did not meet the above-mentioned inclusion criteria were excluded.

Data extraction

All patients' demographic and clinical data were extracted. These included age, gender, comorbidities, and smoking status as demographic characteristics. The patient’s TEVAR-specific clinical data included the grade, presence of other injuries, size and length of the stent, and intraoperative heparinization.

Outcome measures

The demographic variations were explored among the following clinical outcomes for the study participants: technical success rate, subclavian coverage, endo leak, acute limb Ischemia, spinal cord Ischemia, access site complication, and mortality within 30 days.

Statistical analysis

Patients' categorical variables were presented as frequency and percentage. Continuous data were presented as the mean (standard deviation). Logistic regression was utilized to identify predictors of patients’ health status improvement and length of stay (LOS). The IBM SPSS Statistics for Windows, Version 27.0 (Released 2020; IBM Corp., Armonk, New York, United States) was used to conduct the statistical analyses for this study.

Ethical approval

The study protocol was reviewed, and ethical approval was granted by the Research Ethics Committee at the Saudi Ministry of Health in Saudi Arabia (Reference No: H-02-K076- 0922-801). All participants provided their informed consent before participating in the study.

## Results

Patients’ baseline characteristics

A total of 80 patients were involved. The mean age of the patients was 39.0 (standard deviation: 14.3) years. The vast majority of the patients were males (90.0%; n=72). Around 13.8% (n=11) of the patients were smokers. Around 50.0% (n=40) of the patients had grade 3 thoracic aortic injuries. Hypertension was the most common comorbidity among the patients accounting for 15.0% (n=12). The vast majority of the patients (88.8%; n=71) were using heparin. Further details on the baseline characteristics of the patients are provided in Table 1.

**Table 1:** Patients’ baseline characteristics Categorical data have been represented as frequencies and percentages. Continuous data have been represented as mean ± standard deviation.

Variable	Frequency	Percentage
Age (mean (standard deviation) years)	39.0 (14.3) years	
Gender		
Males	72	90.0%
Smoker (Yes)	11	13.8%
Thoracic Aortic Injury Grade		
Grade 1	7	8.8%
Grade 2	33	41.3%
Grade 3	40	50.0%
Comorbidities		
Hypertension	12	15.0%
Diabetes mellitus	6	7.5%
Heart diseases	1	1.3%
Medications use history		
Heparin	71	88.8%
B-blocker	32	40.0%
Corticosteroids	11	13.8%
Angiotensin-converting enzymes	10	12.5%
Calcium channel blocker	9	11.3%
Aspirin	8	10.0%
Antidiabetic agents and insulin	5	6.3%
Anticoagulant (warfarin)	4	5.0%
Antiplatelet	2	2.5%
Statin	1	1.3%

Patients' admission profiles and clinical outcomes

Table 2 presents the admission profiles and clinical outcomes of patients who underwent TEVAR. The median duration of stay was 14.00 days (interquartile range (IQR) 21.00). Only one patient developed post-procedure complications (1.3%). More than half of the patients (62.5%; n=50) developed anemia. Almost one-third (31.3%; n=25) of the patients required subclavian coverage. One patient developed intraoperative endoleak (1.3%). One patient developed an access site complication (1.3%). The mortality rate within 30 days of the operation was 1.3% (n=1). The vast majority of the patients (92.5%; n=74) showed improvement upon discharge from the hospital.

**Table 2:** Patients' admission profiles and clinical outcomes Categorical data have been represented as frequencies and percentages.

Variable	Frequency	Percentage
Duration of stay (median number of days (interquartile range))	14.00 (21.00) days	
Procedures complications (Yes)	1	1.3%
Anemia (Yes)	50	62.5%
Patients clinical outcomes		
Subclavian coverage	25	31.3%
Intraoperative endoleak	1	1.3%
Access site complication (open and percutaneous)	1	1.3%
Mortality (within 30 days)	1	1.3%
Status on discharge		
Discharge against medical advice	2	2.5%
Transferred	3	3.8%
Improved	74	92.5%
Dead	1	1.3%

Predictors of improvement after TEVAR and LOS

Binary logistic regression analysis revealed that baseline patient characteristics and length of hospitalization had no effect on the improvement of patient status upon discharge or their LOS (p<0.05), Table 3.

**Table 3:** Binary logistic regression analysis

Variable	Odds ratio of improvement (95% confidence interval)	P-value	Odds ratio of longer duration of stay (95% confidence interval)	P-value
Age				
Less than 39.0 years (Reference category)	1.00		1.00	
39.0 years and above	0.13 (0.01-1.16)	0.068	1.82 (0.57-3.37)	0.477
Gender				
Females (Reference category)	1.00		1.00	
Males	-		1.86 (0.41-8.38)	0.418
Smoker				
No (Reference category)	1.00		1.00	
Yes	0.78 (0.08-7.40)	0.830	1.80 (0.48-6.72)	0.381
Thoracic Aortic Injury Grade				
Grade 1 (Reference category)	1.00		1.00	
Grade 2	0.68 (0.13-3.61)	0.652	0.69 (0.14-3.31)	0.643
Grade 3	0.85 (0.16-4.49)	0.848	0.67 (0.28-1.65)	0.386
Comorbidities				
Diabetes mellitus	0.36 (0.04-3.73)	0.393	2.00 (0.35-11.60)	0.440
Hypertension	0.32 (0.05-1.97)	0.218	0.91 (0.27-3.13)	0.886
Heart diseases	-		-	
Duration of stay				
Less than 14 days (Reference category)	1.00		N/A	
14 days and over	5.88 (0.66-52.83)	0.114		

## Discussion

This study explores the clinical outcomes of patients who underwent TEVAR including the technical success rate, post-procedural complications, and patients’ mortality within 30 days after the TEVAR procedure. In our study, the median LOS in the hospital for BTAI patients who received TEVAR was 14 days, with an IQR of 21 days. The wide IQR indicates significant variation in the LOS across the study sample. Similar findings have been reported by Mohapatra et al. [20], who found that TEVAR treatment for BTAI patients resulted in a median LOS of 15 days (IQR 8-24). Other studies reported a shorter median LOS of 8 days [21] and a longer median LOS of 27 days (range from three days to 146 days) [22]. These disparities may be attributed to several factors, including variations in patient characteristics, BTAI categories, the presence of complications, and the need for additional treatments or interventions. Moreover, previous studies have shown that delayed TEVAR for BTAI patients resulted in significantly decreased mortality rates [23] but prolonged ICU stays due to related comorbidities [4]. Consequently, patients will need to be monitored for an extended time after delayed TEVAR to evaluate the effectiveness of the intervention and manage any complications that may occur during recovery. This could account for the extended median LOS observed in our findings.

It is necessary to consider the effect of LOS on healthcare resource utilization. Prolonged hospital stays increase healthcare expenses and strain hospital resources, such as beds and medical staff [24-27]. Consequently, it is essential to concentrate on enhancing patient care pathways, optimizing postoperative recovery procedures, and minimizing complications. These efforts will help reduce LOS and ensure efficient resource allocation [28]. A previous review article found that clinical pathways are linked to positive outcomes (such as decreased complications among in-hospital patients) without raising hospital expenses or LOS [29].

One of the notable findings of this study is the low incidence of post-procedure complications following TEVAR in patients with BTAI; only one patient (1.3%) developed complications. This finding highlights the overall safety and effectiveness of TEVAR in managing BTAI patients. Based on a previous study, the TEVAR procedure has been demonstrated to be an effective and safe choice for treating Grade 2 and Grade 3 BTAI patients [30]. Our study is consistent with these findings, as most patients involved in this study also had Grade 2 and Grade 3 BTAI. Given that the TEVAR procedure is a safe and efficient method for these patients, it explains the development of post-procedure complications in only one patient.

Anemia is defined by the WHO as having a hemoglobin (Hgb) level below 12 g/dL for women and below 13 g/dL for men [31]. Following major surgical procedures, post-operative anemia prevalence ranges from 80% to 90% [32]. In our study, more than half of the patients (62.5%) developed anemia following TEVAR for BTAI. Numerous reasons can contribute to postoperative anemia. It can arise from existing anemia before surgery or blood loss due to surgery or trauma [33]. Post-operative anemia is linked to negative consequences, including prolonged hospital stays, mortality, circulatory overload, and infections [34].

Another possible contributing factor to the development of anemia in more than half of the patients in our study is the use of heparin, which can result in bleeding and, consequently, anemia. The SVS issued guidelines for the endovascular repair of traumatic thoracic aortic injuries in 2011 [4]. Given that in the majority of cases, TEVAR can be conducted quickly and holds a low risk of thrombotic complications, some experts have communicated support for performing TEVAR without heparin administration [4]. Nevertheless, the final decision lies with the surgeon, who must carefully evaluate the potential risks of thromboembolic consequences and bleeding before proceeding [4]. Besides, a previous study concluded that the decision to administer heparin in critically ill patients with BTAI undergoing TEVAR is left to the surgeon's discretion, with performing TEVAR without using heparin serving as a safe alternative [35]. Contrary to this, other studies have found that utilizing heparin during TEVAR for BTAI is safe and was not associated with increased hemorrhagic complications [36,37]. We recommend considering the benefits and risks of using heparin for each case before decision-making.

The left subclavian artery (LSA) coverage is usually required to acquire an adequate landing zone [38]. Our findings demonstrate that a notable proportion of patients undergoing TEVAR for BTAI required subclavian artery coverage. Almost one-third of patients (31.3%) in our study required this procedure. Our results are consistent with previous research, suggesting that subclavian artery coverage is commonly needed during TEVAR for BTAI. Approximately 26% to 40% of patients who undergo TEVAR may necessitate coverage of the LSA [39, 40]. In previous studies, the LSA was found to be covered during TEVAR for BTAI in 65.8% [41], 63% [42], and 50% [43] of patients.

Coverage of the LSA during TEVAR can result in various complications, including ischemia in the left upper extremity, spinal cord ischemia, and stroke [44]. Due to the potential risks associated with this procedure and its potential impact on patient outcomes and postoperative complications, it is necessary to conduct a careful preoperative evaluation to avoid complications.

Even though TEVAR is a novel procedure compared with open surgery, serious complications can arise. These complications can be divided into two primary types: ischemic complications resulting from embolic events (such as spinal cord-related ischemic injury, paraplegia, and stroke) and device-related complications (such as stent graft migration and endoleak) [45,46]. Endoleak continues to be a frequently encountered complication and a primary cause for subsequent interventions after TEVAR [47]. Endoleak is characterized by a persistent flow of blood inside the aneurysmal sac and outside the stent graft resulting from the incapacity to establish a secure seal between the stent graft and the aortic wall [48]. In our patient cohort, only one patient developed intraoperative endoleak (1.3%). Previous publications have reported endoleak incidence rates following TEVAR ranging from 5% to 20% [49-56]. Although the occurrence of intraoperative endoleak (1.3%) in our cohort is relatively low, it is crucial to emphasize that endoleak remains a lifelong risk factor in patients undergoing TEVAR, necessitating persistent follow-up [57]. Nevertheless, it is noteworthy that the incidence of endoleak after TEVAR has demonstrated a declining trend over time [38].

Access site complications include pseudoaneurysm, occlusion, rupture, wound infection, hematoma, arteriovenous fistulas, dissection, and arterial thrombosis [38]. Access site complications result in outcomes involving elevated treatment costs, expanded patient mortality and morbidity, and extended hospital stay [58]. Previous studies have reported that access site complications for patients undergoing TEVAR ranged from 10% to 35% [51,59-61]. However, in our cohort (80 patients), only one patient experienced an access site complication (1.3%). This finding could be explained by the fact that 90% of our cohorts were males, while access site complications are more likely to occur among female patients than male patients [62,63]. Furthermore, the findings imply that the treatment approach applied to our cohort might have significantly reduced access site complications during the TEVAR procedure. Over the past few years, evidence has indicated that the routine adoption of ultrasound-guided access in endovascular procedures has led to a decline in the rate of access site complications [50,64]. In endovascular aortic repair, closure devices are used to seal percutaneous access sites. Numerous studies have provided evidence of the effectiveness and safety of closure devices [65-67].

In our study on TEVAR for blunt BTAI, one of the critical outcomes assessed was the 30-day mortality rate following the operation. Our findings revealed a low 30-day mortality rate of 1.3% (1/80) among patients who underwent TEVAR for BTAI. These results highlight the effectiveness and safety of TEVAR as a feasible treatment option for BTAI. The overall favorable outcome aligns with a previous study investigating TEVAR in BTAI patients, which similarly reported a low 30-day mortality rate of 2% (1/50) [68]. The low 30-day mortality rate can be attributed to several factors, most notably the minimally invasive nature of the TEVAR procedure [69]. In comparison to open aortic surgery, TEVAR demonstrates significantly lower mortality and morbidity rates [38,69].

This study has limitations. The cross-sectional study design limited our ability to follow-up with the patients and examine causality between the study variables. This is a single-center study, which might have affected the generalizability of the study findings.

## Conclusions

TEVAR demonstrated a high success rate among patients with BTAI accompanied by a low rate of complications. Our investigation reveals that neither baseline patient characteristics nor length of hospitalization significantly impact patient outcomes upon discharge or their LOS. These findings underscore the need for further exploration into factors influencing patient progress, facilitating more targeted interventions and resource allocation in healthcare settings. Further studies with larger sample sizes are required to identify predictors of procedure success rate and LOS. This can help in the development of preventive procedures to improve patients’ outcomes.
